# Susceptibility to Breast Cancer Misinformation Among Chinese Patients: Cross-sectional Study

**DOI:** 10.2196/42782

**Published:** 2023-04-05

**Authors:** Yi Shan, Meng Ji, Zhaoquan Xing, Zhaogang Dong, Xiaofei Xu

**Affiliations:** 1 School of Foreign Studies, Nantong University Nantong China; 2 School of Languages and Cultures, The University of Sydney Sydney Australia; 3 Department of Urology Qilu Hospital of Shandong University Ji'nan China; 4 Department of Clinical Laboratory Qilu Hospital of Shandong University Ji'nan China; 5 Department of Obstetrics and Gynecology Center for Reproductive Medicine Qilu Hospital of Shandong University Ji'nan China

**Keywords:** susceptibility, breast cancer misinformation, Chinese patients, logistic regression, predicting factors, cancer, misinformation, China, breast cancer, policy, age, gender, education, literacy, clinical

## Abstract

**Background:**

Currently, breast cancer is the most commonly diagnosed cancer and the sixth-leading cause of cancer-related deaths among Chinese women. Worse still, misinformation contributes to the aggravation of the breast cancer burden in China. There is a pressing need to investigate the susceptibility to breast cancer misinformation among Chinese patients. However, no study has been performed in this respect.

**Objective:**

This study aims to ascertain whether some demographics (age, gender, and education), some health literacy skills, and the internal locus of control are significantly associated with the susceptibility to misinformation about all types of breast cancers among randomly sampled Chinese patients of both genders in order to provide insightful implications for clinical practice, health education, medical research, and health policy making.

**Methods:**

We first designed a questionnaire comprising 4 sections of information: age, gender, and education (section 1); self-assessed disease knowledge (section 2); the All Aspects of Health Literacy Scale (AAHLS), the eHealth Literacy Scale (eHEALS), the 6-item General Health Numeracy Test (GHNT-6), and the “Internal” subscale of the Multidimensional Health Locus of Control (MHLC) scales (section 3); and 10 breast cancer myths collected from some officially registered and authenticated websites (section 4). Subsequently, we recruited patients from Qilu Hospital of Shandong University, China, using randomized sampling. The questionnaire was administered via *wenjuanxing*, the most popular online survey platform in China. The collected data were manipulated in a Microsoft Excel file. We manually checked the validity of each questionnaire using the predefined validity criterion. After that, we coded all valid questionnaires according to the predefined coding scheme, based on Likert scales of different point (score) ranges for different sections of the questionnaire. In the subsequent step, we calculated the sums of the subsections of the AAHLS and the sums of the 2 health literacy scales (the eHEALS and GHNT-6) and the 10 breast cancer myths. Finally, we applied logistic regression modeling to relate the scores in section 4 to the scores in sections 1-3 of the questionnaire to identify what significantly contributes to the susceptibility to breast cancer misinformation among Chinese patients.

**Results:**

All 447 questionnaires collected were valid according to the validity criterion. The participants were aged 38.29 (SD 11.52) years on average. The mean score for their education was 3.68 (SD 1.46), implying that their average educational attainment was between year 12 and a diploma (junior college). Of the 447 participants, 348 (77.85%) were women. The mean score for their self-assessed disease knowledge was 2.50 (SD 0.92), indicating that their self-assessed disease knowledge status was between “knowing a lot” and “knowing some.” The mean scores of the subconstructs in the AAHLS were 6.22 (SD 1.34) for functional health literacy, 5.22 (SD 1.54) for communicative health literacy, and 11.19 (SD 1.99) for critical health literacy. The mean score for eHealth literacy was 24.21 (SD 5.49). The mean score for the 6 questions in the GHNT-6 was 1.57 (SD 0.49), 1.21 (SD 0.41), 1.24 (SD 0.43), 1.90 (SD 0.30), 1.82 (SD 0.39), and 1.73 (SD 0.44), respectively. The mean score for the patients’ health beliefs and self-confidence was 21.19 (SD 5.63). The mean score for their response to each myth ranged from 1.24 (SD 0.43) to 1.67 (SD 0.47), and the mean score for responses to the 10 myths was 14.03 (SD 1.78). Through interpreting these descriptive statistics, we found that Chinese female patients’ limited ability to rebut breast cancer misinformation is mainly attributed to 5 factors: (1) lower communicative health literacy, (2) certainty about self-assessed eHealth literacy skills, (3) lower general health numeracy, (4) positive self-assessment of general disease knowledge, and (5) more negative health beliefs and lower levels of self-confidence.

**Conclusions:**

Drawing on logistic regression modeling, we studied the susceptibility to breast cancer misinformation among Chinese patients. The predicting factors of the susceptibility to breast cancer misinformation identified in this study can provide insightful implications for clinical practice, health education, medical research, and health policy making.

## Introduction

### Background

Breast cancer is the most commonly diagnosed cancer in females worldwide, and it is the leading cause of cancer-induced deaths among females [1]. More than 1 million people are diagnosed with breast cancer across the world annually, and at least 400,000 women die of this disease throughout the world each year, representing 14% of the total cancer-caused deaths, as estimated in previous studies [2-4]. The highest breast cancer incidence and prevalence rates were reported in North America, Australia, New Zealand, and Northern and Western Europe, whereas the lowest were reported in East Asia [5]. In contrast, there was a low breast cancer incidence in China, but the rate in China has grown more than twice as fast as the global rate, particularly in urban areas, since the 1990s [6]. According to Ferlay et al [6], the age-standardized breast cancer rate per 100 000 women is 21.6 cases. It was predicted that the breast cancer incidence in China was likely to increase from <60 cases per 100 000 to >100 cases per 100 000 among women aged 55-69 years and reach 2.5 million cases by 2021 [7]. Currently, breast cancer is the most commonly diagnosed cancer and the sixth-leading cause of cancer deaths among women in China [8].

Misinformation contributes to the aggravation of the breast cancer burden in China. As found in previous studies, health misinformation hinders the delivery of evidence-based medicine, influences patient-physician relationships negatively, and causes increased health risks [9-12]. Misinformation is, therefore, deemed a global risk [13]. This is especially true for cancer care, as the uptake of unverified therapies is closely associated with reduced survival chances [12]. After reading misinformation, cancer patients live in the hope in many cases that a flawed and potentially harmful, even deadly, untested therapy could cure their disease [14]. Accordingly, they might most likely act upon misinformation, resulting in wrong medical decisions and disastrous health outcomes.

Cancer misinformation is highly prevalent in China, as in other countries. There was an approximate 1 in 3 chance for patients to cite misinformation on cancer diagnoses and treatments during clinical appointments with their doctors [15]. Given this high prevalence of misinformation, Johnson et al [15] explored misinformation about the 4 most common cancers (ie, breast cancer, prostate cancer, colorectal cancer, and lung cancer). They concluded that of the 200 papers investigated, 65 (32.5%) contained misinformation and 61 (30.5%) contained harmful information and that among the papers containing misinformation, 50 (76.9%) of 65 contained harmful information [15]. Other studies have investigated the nature and spread of social media–based cancer misinformation [16], including false elements possibly distorting people’s attitudes toward and actions on cancer prevention and treatment [16]; misleading perceptions of the causes, prevention, and treatment of cancers [17]; and the veracity of information [18]. To make things worse, social media has exacerbated individuals’ uncertainties about cancers, making them most likely to be led astray by cancer misinformation [16]. In this scenario, there is a pressing need to ascertain specific factors contributing to patients’ susceptibility to breast cancer misinformation, to “improve the reach of high quality health information among people with limited health literacy and thereby increase the effectiveness of health communication programs and campaigns” [19]. However, no study has been performed in this respect based on our search and analysis of the relevant literature.

The relationship between health literacy and information seeking and information processing has been well documented in the literature. Health literacy has been defined as the ability to obtain, process, and understand basic health information and services essential for making appropriate health decisions [20]. Individuals with suboptimal health literacy have more difficulty understanding and applying health information compared to people with adequate health literacy [21]. The process of using information has been identified as a key component of health literacy, and compared with individuals with adequate health literacy, those with limited health literacy experience more barriers to using accurate health information [19,22]. Four core competencies have been found to contribute to health literacy, including the ability to access (seeking, finding, and obtaining), understand, apply, and appraise health information [23]. Informed by these studies, we hypothesized that individuals’ health literacy is closely related to their susceptibility to health-related misinformation. However, few studies have been conducted to explore the contribution of health literacy to misinformation vulnerability according to our examination of the literature. For example, Khan and Idris found [24] that the perceived self-efficacy to detect misinformation on social media could be predicted by internet skills of health information seeking and verification. This gap in the literature needs to be filled.

Some studies, though few, have investigated the relationship between health misinformation susceptibility and participants’ demographics. Khan and Idris [24] also found that the perceived self-efficacy to detect misinformation on social media could be predicted by the level of education. The extant literature has well documented the relationship between health literacy and demographic characteristics, including age, gender, and education. For example, lower health literacy was found to be correlated with increasing age [25,26], the female gender [27], and lower educational attainment [27,28]. Based on these ascertained associations between some demographic features and health literacy and informed by some potential relationship between health literacy and misinformation susceptibility reported by Khan and Idris [24], we hypothesized that such demographics as age, gender, and education might be correlated with misinformation susceptibility. However, such hypothesized correlations need to be further ascertained to fill the gap in the literature.

In addition, individuals’ internal locus of control, that is, “beliefs that the source of reinforcements for health-related behaviors is primarily internal” [29], can motivate people to take voluntary action to promote health and reduce health risks [30], mediate health status [31,32], engage in health behaviors, and become more knowledgeable about their health problems [33,34]. Informed by these findings, we hypothesized that people’s internal locus of control may be related to health misinformation susceptibility. However, relevant studies need to be conducted to verify this hypothesis to fill the gap in the literature.

### Objective

Based on the problems we identified and the hypotheses we proposed, this study aims to ascertain whether some demographics (age, gender, and education), some health literacy skills, and the internal locus of control are significantly associated with the susceptibility to misinformation about all types of breast cancers among randomly sampled Chinese patients of both genders in order to provide insightful implications for clinical practice, health education, medical research, and health policy making.

## Methods

### Questionnaire Design

A 4-section questionnaire was designed to elicit information essential for the study objective from Chinese patients. Section 1 was related to participants’ information about age, gender, and education. In section 2, the participants were asked to self-assess their disease knowledge. Section 3 assessed the participants’ health literacy skills using 3 validated health literacy instruments: the All Aspects of Health Literacy Scale (AAHLS) [35], the eHealth Literacy Scale (eHEALS) [36], and the 6-item General Health Numeracy Test (GHNT-6) [37]. The 12-item AAHLS contains 3 subscales: the communicative health literacy subscale, the functional health literacy subscale, and the critical health literacy subscale. The 8-item eHEALS evaluates the informants’ knowledge and skills that are essential for using eHealth resources and interventions. The GHNT-6 assesses study participants’ understanding and capacities to act upon numerical health information to help health providers and educators tailor education to patients. Both eHEALS and the GHNT-6 do not have subscales. Meanwhile, this section assessed the participants’ internal locus of control using the “Internal” subscale of the Multidimensional Health Locus of Control (MHLC) scales [38] that has been validated in previous studies [29,31-34]. Section 4 was designed to test the participants’ susceptibility to breast cancer misinformation using 10 myths about breast cancer collected from some officially registered and authenticated websites (Multimedia Appendix 1) [39-41]. Informed by relevant studies [20-34], we hypothesized that the information collected in sections 1-3 is expected to correlate with the participants’ susceptibility to breast cancer myths listed in section 4.

As mentioned before, the AAHLS, eHEALS, GHNT-6, and MHLC are validated scales. The remaining sections (1, 2, and 4) were validated by 3 researchers (authors ZD, ZX, and XX) from the perspectives of clinical practice and health education. As such, we could ensure that our questionnaire was valid.

### Informant Recruitment and Online Survey

Patients were recruited from Qilu Hospital of Shandong University, China, through randomized sampling. The participant inclusion criteria included (1) being aged 18 years or older, (2) having at least primary education to understand the questionnaire items, (3) being unique patients distinguished by their unique questionnaire IDs, and (4) voluntarily participating in the survey. We made face-to-face contact with Chinese patients attending the outpatient clinic of Qilu Hospital and those being hospitalized there in order to identify those who satisfied the inclusion criteria, tell them the purpose of the survey, and ask them to participate in the online survey as scheduled. In this stage, we approached 488 eligible patients.

The questionnaire was administered via *wenjuanxing* [42], the most popular online survey platform in China, from July 20 to August 20, 2022. According to our predefined criterion, a returned questionnaire was valid only when all questions involved in it were answered.

### Data Collection, Coding, and Analysis

On August 21, 2022, the data were collected from *wenjuanxing* and stored in a Microsoft Excel file. A total of 447 answered questionnaires were returned, with a response rate of 92% (447/488). We double-checked the returned questionnaires, finding all of them valid. After that, we coded all valid questionnaires according to the predefined coding schemes, which were based on Likert scales of different point (score) ranges for different sections of the questionnaire. Subsequently, we calculated the sums of the subsections of the AAHLS and the sums of the 2 health literacy scales (eHEALS and the GHNT-6), the “Internal” subscale of the MHLC, and the 10 breast cancer myths. Finally, we applied logistic regression modeling to relate the sums in section 4 (dependent variables) to the sums in sections 1-3 (independent variables) to identify factors significantly associated with the susceptibility to breast cancer misinformation among Chinese patients.

We set the cutoff score for breast cancer misinformation susceptibility at 5 correct answers to the 10 myths about breast cancer. Specifically, if the study participants returned 5 or fewer correct answers to these 10 myths, they were regarded as being susceptible to breast cancer misinformation.

### Ethical Considerations

This study was approved by the ethics review board of Qilu Hospital of Shandong University, China (review number KYLL-202208-026). Written consent was obtained from the participants. The data collected were anonymous or deidentified for privacy and confidentiality protection. We recruited patients who were willing to support our research without compensation.

## Results

### Descriptive Statistics of the Information Collected

The descriptive statistics of the survey participants are presented in Multimedia Appendix 2. All 447 answered questionnaires were valid according to the validity criterion. The participants were aged 38.29 (SD 11.52) years on average. The mean score for their education was 3.68 (SD 1.46), implying that their average educational attainment was between year 12 and a diploma (junior college). Of the 447 participants, 348 (77.85%) were women. The mean score for their self-assessed disease knowledge was 2.50 (SD 0.92), indicating that their self-assessed disease knowledge status was between “knowing a lot” and “knowing some.” The mean scores of the subconstructs in the AAHLS were 6.22 (SD 1.34) for functional health literacy, 5.22 (SD 1.54) for communicative health literacy, and 11.19 (SD 1.99) for critical health literacy. These mean values indicated that the patients “sometimes” relied on help to read health information, they “sometimes” knew the effective ways of communication with health providers, and they were “sometimes” critical about health information, respectively. The mean score for eHealth literacy was 24.21 (SD 5.49), implying the participants’ relatively low level of self-assessed eHealth literacy (ie, they were unsure of their eHealth literacy skills). The mean score for the 6 questions in the GHNT-6 was 1.57 (SD 0.49), 1.21 (SD 0.41), 1.24 (SD 0.43), 1.90 (SD 0.30), 1.82 (SD 0.39), and 1.73 (SD 0.44), respectively. These scores mean that a high proportion of patients responded to the questions incorrectly, especially questions 1, 4, 5, and 6. The mean score (21.19, SD 5.63) for the patients’ health beliefs and self-confidence indicated that their answers to the 6 items of the “Internal” subscale of the MHLC were between “slightly disagree” and “slightly agree.” In other words, they were not self-confident about their self-management of health and disease. The mean score for their response to each myth ranged from 1.24 (SD 0.43) to 1.67 (SD 0.47), and the mean score for responses to the 10 myths was 14.03 (SD 1.78). This implies that a large proportion of participants returned incorrect answers to the 10 myths.

### Multilinearity Statistics of the Predictor Variables

#### Multicollinearity

Collinearity statistics, including the variance inflation factor (VIF) and tolerance, showed that the correlation among the 10 predictor variables was at an acceptable level, as all VIF scores were <2 and their matching tolerance scores were <1 [43,44]; see Table 1. The 10 predictor variables were age, gender, highest educational attainment, self-reported disease knowledge, sum of functional health literacy (FHL_SUM) [35], sum of communicative health literacy (COHL_SUM) [35], sum of critical health literacy (CRHL_SUM) [35], sum of eHealth literacy (eHL_SUM) [36], sum of general health numeracy test (GHNT_SUM) [37], and sum of the 6 “Internal” subscale items on health beliefs and self-confidence in the MHLC scales (MHLC_SUM) [38].

**Table 1:** Collinearity statistics.

Predictor variable	Tolerance	VIF^a^
Age	0.79	1.27
Gender	0.86	1.16
Highest educational attainment	0.70	1.43
Self-assessed disease knowledge	0.94	1.06
FHL_SUM^b^	0.97	1.03
COHL_SUM^c^	0.76	1.31
CRHL_SUM^d^	0.88	1.14
eHL_SUM^e^	0.69	1.45
GHNT_SUM^f^	0.89	1.13
MHLC_SUM^g^	0.79	1.27

^a^VIF: variance inflation factor.

^b^FHL_SUM: sum of functional health literacy.

^c^COHL_SUM: sum of communicative health literacy.

^d^CRHL_SUM: sum of critical health literacy.

^e^eHL_SUM: sum of eHealth literacy.

^f^GHNT_SUM: sum of general health numeracy.

^g^MHLC_SUM: sum of the 6 “Internal” subscale items on health beliefs and self-confidence in the Multidimensional Health Locus of Control (MHLC) scales.

#### Factors Associated With Breast Cancer Misinformation Susceptibility Among Females of All Ages

Table 2 shows the logistic regression modeling result of the responses from female participants of all age groups. First, lower communicative health literacy was a significant predictor of the susceptibility to breast cancer misinformation. When the frequency of the study participant of giving health professionals “all the information they need to help you” dropped from “often” to “sometimes,” the odds of the individual having 5 or fewer correct responses on the questionnaire increased by 111% (COHL1, “sometimes”: odds ratio [OR] 2.11, 95% CI 1.19-3.73, *P*=.01). When the frequency dropped from “often” to “rarely,” the odds of the individual having 5 or fewer correct responses increased by as much as 309% (COHL1, “rarely”: OR 4.09, 95% CI 1.78-9.42, *P*<.001). It was found that the uncertainty about self-reported eHealth literacy skills was associated with a significant decrease in the odds of a participant having 5 or fewer correct responses to the questionnaire on breast cancer misinformation. This uncertainty was reflected in 2 of the 8-item eHEALS (eHL1, “I know what health resources are available on the internet”: OR 0.33, 95% CI 0.14-0.74, *P*=.01; eHL5, “I know how to find helpful health resources on the internet”: OR 0.41, 95% CI 0.17-0.98, *P*=.04). Specifically, this result meant that when an individual reported that they were uncertain about their ability to identify and ascertain the usefulness of online health resources, the odds of the individual having 5 or fewer correct responses decreased by 67% (eHL1) and 59% (eHL5) compared to the reference response “strongly disagree.” There were no statistically significant changes in the number of correct responses when study participants reported they “disagree,” “agree,” or “strongly agree” with questions of eHEALS. Lower general health numeracy also predicted a significant increase in the odds of having 5 or fewer correct responses. Specifically, the last question of the GHNT-6 was a significant predictor of the response outcome: (GHNT_6: OR 2.20, 95% CI 1.14-4.25, *P*=.02). In the SPSS modeling process, the reference class was 1 (correct answer). So, the result indicated that when an individual incorrectly answered the last question of the GHNT-6 (GHNT_6), which was related to the interpretation of breast cancer screening test results, the odds of that individual having 5 or fewer correct answers on the questionnaire of breast cancer misinformation increased by as much as 120%.

**Table 2:** Factors associated with breast cancer misinformation susceptibility (threshold=0.5, female, all ages).

Predictor variable		B	SE	Wald test (*df*)	*P* value	Exp(B) (95% CI)	
**COHL1^a^**							
	COHL1 (reference: often)	N/A^b^	N/A	13.37 (2)	<.001	N/A	
	COHL1 (sometimes)	0.75	0.29	6.62 (1)	.01^c^	2.11^c^ (1.19-3.73)	
	COHL1 (rarely)	1.41	0.43	10.96 (1)	<.001^c^	4.09^c^ (1.78-9.42)	
**eHL1^d^**							
	eHL1 (reference: strongly disagree)	N/A	N/A	9.69 (4)	.05	N/A	
	eHL1 (disagree)	–0.26	0.44	0.36 (1)	.55	0.77 (0.32-1.82)	
	eHL1 (unsure)	–1.12	0.42	7.09 (1)	.01^c^	0.33^c^ (0.14-0.74)	
	eHL1 (agree)	–0.87	0.47	3.38 (1)	.07	0.42 (0.17-1.06)	
	eHL1 (strongly agree)	–0.89	0.66	1.81 (1)	.18	0.41 (0.11-1.50)	
**eHL5**							
	eHL5 (reference: strongly disagree)	N/A	N/A	10.49 (4)	.03	N/A	
	eHL5 (disagree)	0.37	0.50	0.54 (1)	.46	1.45 (0.54-3.86)	
	eHL5 (unsure)	–0.89	0.44	4.07 (1)	.04^c^	0.41^c^ (0.17-0.98)	
	eHL5 (agree)	–0.40	0.45	0.81 (1)	.37	0.67 (0.28-1.61)	
	eHL5 (strongly agree)	–0.14	0.55	0.06 (1)	.80	0.87 (0.29-2.57)	
GHNT_6^e^ (wrong; reference: correct)		0.79	0.34	5.53 (1)	.02^c^	2.20^c^ (1.14-4.25)	
Constant		–0.59	0.57	1.05 (1)	.30	0.55 (N/A)	

^a^COHL: communicative health literacy.

^b^N/A: not applicable.

^c^Predicted membership: 5 or fewer correct answers.

^d^eHL: eHealth literacy.

^e^GHNT: General Health Numeracy Test.

#### Factors Associated With Breast Cancer Misinformation Susceptibility Among Females Aged 40 Years or Over

Table 3 shows the results of regression modeling of the responses from female participants aged 40 years or above. The results showed that for middle-aged or elderly Chinese women, the only significant predictor of their ability to appraise online misinformation about breast cancer was their general health numeracy skill. Specifically, if they had an inaccurate response to the last question of the GHNT-6 (GHNT_6), the odds of having 5 or fewer correct assessments of the 10 misinformation statements increased by 412% (OR 5.12, 95% CI 1.61-16.31, *P*=.01).

**Table 3:** Factors associated with breast cancer misinformation susceptibility (threshold=0.5, female, age≥40 years).

Predictor variable		B	SE	Wald test (*df*)	*P* value	Exp(B) (95% CI)
**CRHL5^a^**						
	CRHL5 (reference: yes)	N/A^b^	N/A	6.58 (2)	.04	N/A
	CRHL5 (maybe)	–0.80	0.47	2.90 (1)	.09	0.45 (0.18-1.13)
	CRHL5 (no)	0.45	0.52	0.75 (1)	.39	1.56 (0.57-4.30)
GHNT_6^c^ (wrong)		1.63	0.59	7.64 (1)	.01^d^	5.12^d^ (1.61-16.31)
Constant		–1.46	0.63	5.38 (1)	.02	0.23 (N/A)

^a^CRHL: critical health literacy.

^b^N/A: not applicable.

^c^GHNT: General Health Numeracy Test.

^d^Predicted membership: 5 or fewer correct answers.

#### Factors Associated With Breast Cancer Misinformation Susceptibility Among Females of All Ages With Education of Year 12 or Below

Table 4 shows the results of logistic regression modeling of responses from Chinese female participants with limited education (ie, having completed up to year 12 schooling). The results showed that when a study participant had more moderate self-assessment of their general disease knowledge, their odds of having 5 or fewer correct assessments of breast cancer misinformation decreased significantly. In the regression modeling process, the reference class of self-assessed disease knowledge was “I know diseases very well.” When the self-assessment level decreased to “I have a lot of disease knowledge,” the odds of having 5 or fewer correct misinformation assessments reduced by 72% (OR 0.28, 95% CI 0.09-0.92, *P*=.04). When the assessment level decreased to “I have some disease knowledge,” the odds of having 5 or fewer correct misinformation assessments reduced by 89% (OR 0.11, 95% CI 0.04-0.35, *P*<.001). Again, low communicative health literacy proved a significant predictor of limited misinformation appraisal ability. It shows that when the frequency of a female “giving all the information that your doctors and nurses need when you talk to them” changed from “often” to “rarely,” the odds of that individual having 5 or fewer correct responses increased by 379% (OR 4.79, 95% CI 1.44-15.88, *P*=.01). Lastly, limited general health numeracy skills predicted worse response outcomes. When a Chinese female with up to year 12 education had an inaccurate response to the last item of the GHNT-6 (GHNT_6), their odds of having 5 or fewer correct responses increased by 1010% (OR 11.10, 95% CI 1.31-94.35, *P*=.03).

**Table 4:** Factors associated with breast cancer misinformation susceptibility (threshold=0.5, female only, all ages; education: year 12 or below).

Predictor variable			B		SE		Wald test (*df*)		*P* value		Exp(B) (95% CI)
**Disease knowledge self-assessed**											
	Disease knowledge self-assessed (reference: very well)	N/A^a^		N/A		14.33 (3)		<.001		N/A	
	Disease knowledge (a lot)	–1.26		0.60		4.40 (1)		.04^b^		0.28^b^ (0.09-0.92)	
	Disease knowledge (some)	–2.19		0.58		14.16 (1)		<.001^b^		0.11^b^ (0.04-0.35)	
	Disease knowledge (little)	–0.96		0.61		2.49 (1)		.11		0.38 (0.12-1.26)	
**COHL1^c^**											
	COHL1 (reference: often)	N/A		N/A		7.18 (2)		.03		N/A	
	COHL1 (sometimes)	0.87		0.46		3.54 (1)		.06		2.39 (0.96-5.95)	
	COHL1 (rarely)	1.57		0.61		6.55 (1)		.01^b^		4.79^b^ (1.44-15.88)	
GHNT_6^d^ (1)			2.41		1.09		4.86 (1)		.03^b^		11.10^b^ (1.31-94.35)
Constant			–1.85		1.16		2.52 (1)		.11		0.16 (N/A)

^a^N/A: not applicable.

^b^Predicted membership: 5 or fewer correct answers.

^c^COHL: communicative health literacy.

^d^GHNT: General Health Numeracy Test.

#### Factors Associated With Breast Cancer Misinformation Susceptibility Among Females of All Ages With Education Above Year 12

Table 5 shows the results of the logistic regression modeling of responses from Chinese female participants with an educational level higher than year 12. The results showed that if an individual in this group reported that she was unsure about “what health resources are available on the internet” (eHL1), their odds of having 5 or fewer correct responses decreased by 87% compared to females who reported “strongly disagree” (eHL1, “unsure”: OR 0.13, 95% CI 0.04-0.48, *P*<.001). There were no significant changes in the number of correct responses to the misinformation questionnaire when the participants returned other responses to eHL1, such as “disagree” (*P*=.08), “agree” (*P*=.55), or “strongly agree” (*P*=.06). When a female participant in the better-educated group reported that she “disagreed” with the statement “I know where to find helpful health resources on the internet,” their odds of having 5 or fewer correct responses to the breast cancer misinformation questionnaire increased by 1123%: (eHL4, “disagreed”: OR 0.13, 95% CI 0.04-0.48, *P*<.001). There were no significant changes in the number of correct responses to the misinformation questionnaire when the participants returned other responses to eHL4, such as “unsure” (*P*=.20), “agree” (*P*=.84), or “strongly agree” (*P*=.06). Lower general health numeracy predicted limited breast cancer misinformation ability. Among female participants in the better-educated group, when an individual had an inaccurate answer to item 5 of the GHNT-6 about the probability of heart attacks after taking cholesterol medicine, their odds of having 5 or fewer correct answers to the breast cancer misinformation questionnaire increased by 189% (GHNT_5: OR 2.89, 95% CI 1.19-7.04, *P*=.02).

**Table 5:** Factors associated with breast cancer misinformation susceptibility (threshold=0.5, female only, all ages; education: above year 12).

Predictor variable		B	SE	Wald test (*df*)	*P* value	Exp(B) (95% CI)
FHL_SUM^a^		–0.28	0.13	4.22 (1)	.04	0.76 (0.58-0.99)
**eHL1^b^**						
	eHL1 (reference: strongly disagree)	N/A^c^	N/A	13.18 (4)	.01	N/A
	eHL1 (disagree)	–1.26	0.73	3.02 (1)	.08	0.28 (0.07-1.17)
	eHL1 (unsure)	–2.04	0.66	9.48 (1)	<.001^d^	0.13^d^ (0.04-0.48)
	eHL1 (agree)	–0.60	0.65	0.84 (1)	.36	0.55 (0.15-1.98)
	eHL1 (strongly agree)	–2.08	1.12	3.44 (1)	.06	0.13 (0.01-1.13)
**eHL4**						
	eHL4 (reference: strongly disagree)	N/A	N/A	18.29 (4)	<.001	N/A
	eHL4 (disagree)	2.50	0.93	7.19 (1)	.01^d^	12.23^d^ (1.96-76.22)
	eHL4 (unsure)	1.16	0.91	1.63 (1)	.20	3.19 (0.54-18.96)
	eHL4 (agree)	0.18	0.92	0.04 (1)	.84	1.20 (0.20-7.26)
	eHL4 (strongly agree)	2.32	1.21	3.67 (1)	.06	10.16 (0.95-108.74)
GHNT_5^e^ (wrong)		1.06	0.45	5.48 (1)	.02^d^	2.89^d^ (1.19-7.04)
Constant		–0.20	1.26	0.02 (1)	.88	0.82 (N/A)

^a^FHL_SUM: sum of functional health literacy.

^b^eHL: eHealth literacy.

^c^N/A: not applicable.

^d^Predicted membership: 5 or fewer correct answers.

^e^GHNT: General Health Numeracy Test.

#### Health Beliefs Associated With Breast Cancer Misinformation Susceptibility Among Participants Regardless of Age, Gender, and Education

Next, we examined the relationship between health belief patterns and the participants’ ability to appraise online misinformation about breast cancer, as shown in Table 6. Since breast cancer is not limited to females, our analysis included Chinese male participants. We selected questions from MHLC Form A and administered the questionnaire to both female and male participants. It was found that among participants of both genders, statement 6 of MHLC Form A (MHLC_A6: “I am in control of my health”) was a significant predictor. We used “strongly disagree” as the reference category and found that when a participant reported “slightly agree,” their odds of having 5 or fewer correct responses to the breast cancer misinformation list decreased by 73% (MHLC_A6, “slightly agree”: OR 0.27, 95% CI 0.13-0.57, *P*<.001). When a participant reported “moderately agree,” their odds of having 5 or fewer correct responses to the breast cancer misinformation list decreased by 72% (MHLC_A6, “moderately agree”: OR 0.28, 95% CI 0.14-0.58, *P*<.001). When a participant reported “strongly agree,” their odds of having 5 or fewer correct responses to the breast cancer misinformation list decreased by 69% (MHLC_A6, “strongly agree”: OR 0.31, 95% CI 0.15-0.63, *P*<.001).

**Table 6:** Health beliefs associated with breast cancer misinformation susceptibility (threshold=0.5, both genders, all ages, all education levels).

Predictor variable		B	SE	Wald test (*df*)	*P* value	Exp(B) (95% CI)	
**MHLC_A6^a^**							
	MHLC_A6 (reference: strongly disagree)	N/A^b^	N/A	22.59 (5)	<.001	N/A	
	MHLC_A6 (moderately disagree)	–0.39	0.38	1.02 (1)	0.31	0.68 (0.32-1.44)	
	MHLC_A6 (slightly disagree)	–0.53	0.38	1.99 (1)	0.16	0.59 (0.28-1.23)	
	MHLC_A6 (slightly agree)	–1.31	0.38	11.86 (1)	<.001^c^	0.27^c^ (0.13-0.57)	
	MHLC_A6 (moderately agree)	–1.27	0.37	11.89 (1)	<.001^c^	0.28^c^ (0.14-0.58)	
	MHLC_A6 (strongly agree)	–1.18	0.36	10.48 (1)	<.001^c^	0.31^c^ (0.15-0.63)	
Constant		0.32	0.29	1.27 (1)	0.26	1.38 (N/A)	

^a^MHLC: Multidimensional Health Locus of Control.

^b^N/A: not applicable.

^c^Predicted membership: 5 or fewer correct answers.

#### Health Beliefs Associated With Breast Cancer Misinformation Susceptibility Among Participants Among Females Regardless of Age and Education

Table 7 shows the relationship between health beliefs and breast cancer misinformation appraisal ability among Chinese female participants. The results showed that statement 6 of MHLC Form A (MHLC_A6: “I am in control of my health”) and statement 12 (MHLC_A12: “The main thing that affects my health is what I myself do”) were strongly associated with the response outcome. First, using the response “strongly disagree” as the reference category, when a participant reported “slightly agree” to MHLC_A6, their odds of having 5 or fewer correct responses to the breast cancer misinformation list decreased by 72% (MHLC_A6, “slightly agree”: OR 0.28, 95% CI 0.14-0.58, *P*<.001). The odds continued to decrease by 73% when a participant reported “moderately agree” (MHLC_A6, “moderately agree”: OR 0.27, 95% CI 0.11-0.66, *P*<.001) and decrease by 74% when a participant reported “strongly agree” (MHLC_A6, “strongly agree”: OR 0.26, 95% CI 0.10-0.66, *P*<.001). Second, using the “strongly disagree” as the reference category, when a participant reported “slightly agree” to MHLC_A12, their odds of having 5 or fewer correct responses to the breast cancer misinformation list decreased by 72% (MHLC_A12, “slightly agree”: OR 0.28, 95% CI 0.10-0.77, *P*=.01). The odds continued to decrease by 68% when a participant reported “moderately agree” (MHLC_A12, “moderately agree”: OR 0.32, 95% CI 0.12-0.85, *P*=.02), and decrease by 65% when a participant reported “strongly agree” (MHLC_A12, “strongly agree”: OR 0.35, 95% CI 0.12-0.97, *P*=.04).

**Table 7:** Health beliefs associated with breast cancer misinformation susceptibility (threshold=0.5, females, all ages, all education levels).

Predictor variable			B	SE		Wald test (*df*)		*P* value		Exp(B) (95% CI)
**MHLC_A6^a^**										
	MHLC_A6 (reference: strongly disagree)	N/A^b^		N/A	13.09 (5)		.02		N/A	
	MHLC_A6 (moderately disagree)	–0.45		0.51	0.78 (1)		.38		0.64 (0.23-1.73)	
	MHLC_A6 (slightly disagree)	–0.79		0.48	2.76 (1)		.10		0.45 (0.18-1.15)	
	MHLC_A6 (slightly agree)	–1.29		0.48	7.27 (1)		.01^c^		0.28^c^ (0.11-0.70)	
	MHLC_A6 (moderately agree)	–1.33		0.47	8.12 (1)		<.001^c^		0.27^c^ (0.11-0.66)	
	MHLC_A6 (strongly agree)	–1.36		0.48	7.99 (1)		<.001^c^		0.26^c^ (0.10-0.66)	
**MHLC_A12**										
	MHLC_A12 (reference: strongly disagree)	N/A		N/A	11.66 (5)		.04		N/A	
	MHLC_A12 (moderately disagree)	–0.38		0.51	0.58 (1)		.45		0.68 (0.25-1.84)	
	MHLC_A12 (slightly agree)	–1.29		0.52	6.11 (1)		.01^c^		0.28^c^ (0.10-0.77)	
	MHLC_A12 (moderately agree)	–1.14		0.50	5.26 (1)		.02^c^		0.32^c^ (0.12-0.85)	
	MHLC_A12 (strongly agree)	–1.06		0.52	4.07 (1)		.04^c^		0.35^c^ (0.12-0.97)	
	MHLC_A12 (slightly disagree)	–0.49		0.59	0.69 (1)		.41		0.61 (0.19-1.94)	
Constant			1.15	0.54		4.51 (1)		.03		3.16 (N/A)

^a^MHLC: Multidimensional Health Locus of Control.

^b^N/A: not applicable.

^c^Predicted membership: 5 or fewer correct answers.

## Discussion

### Principal Findings

Using logistic regression modeling, we investigated the susceptibility to breast cancer misinformation among Chinese patients. It was found that 5 factors contributed significantly to the limited ability to appraise breast cancer misinformation, especially among female study participants, as reported in the following sections.

#### Principal Finding 1: Lower Communicative Health Literacy Significantly Predicted Female Patients’ Susceptibility to Breast Cancer Misinformation

We identified low communicative health literacy as a significant predictor of female participants’ limited breast cancer misinformation appraisal ability. This finding confirms some findings reported in a few previous studies [23,45-50]. Freebody and Luke [46] proposed interactive (communicative) health literacy (ie, cognitive and literacy skills used to actively participate in daily activities and apply new information to changing situations) as 1 of the 3 subsets of skills comprising health literacy skills. Like functional health literacy and critical health literacy, communicative health literacy has been found to allow people to navigate the domains of health care, disease prevention, and health promotion [23]. Adult individuals with higher health literacy are more likely to seek health information from the internet and their health care provider, as found by Gaglio et al [47] and Sheih et al [48] in their studies. It follows that people with higher communicative health literacy are more likely to have higher probabilities of being equipped with adequate or high breast cancer health literacy. In contrast, individuals with poor communicative health literacy tend to have higher probabilities of having limited breast cancer health literacy, as reported in our study. Several previous studies have reported similar findings concerning the correlation of health literacy with cancer-related attitudes, knowledge, and behaviors [45,49,50].

#### Principal Finding 2: Uncertainty About Self-Assessed eHealth Literacy Skills Was a Predictor of Female Participants’ Higher Levels of Abilities to Rebut Breast Cancer Misinformation

Interestingly, this finding is not in tune with some findings in the literature. Contrary to some previous studies [51-56], we found in our study that female patients are probably more capable to appraise and rebut breast cancer misinformation when reporting uncertainty about their ability to identify and ascertain the usefulness of online health resources. Specifically, uncertainty about 2 items from the 8-item eHEALS (item 1, “I know what health resources are available on the internet,” and item 5, “I know how to find helpful health resources on the internet”) predicted lower odds of susceptibility to breast cancer misinformation among women patients regardless of age and education. Similarly, uncertainty about item 1 (“I know what health resources are available on the internet”) significantly predicted that well-educated female patients (with above year 12 schooling) had lower probabilities of vulnerability to breast cancer misinformation. However, women in this better-educated group were far more probably susceptible to breast cancer misinformation when reporting uncertainty about item 4 (“I know where to find helpful health resources on the internet”) of eHEALS.

However, some previous studies have reported different findings. According to Jensen et al [51], individuals increasingly adopting eHealth services are more likely to properly care and manage their own conditions using eHealth. People with higher levels of eHealth literacy, a set of knowledge and skills necessary for interacting with technology-based health tools productively [52], could more likely fully use health services and information that are delivered or enhanced via the internet and related technologies [53], including patient education, remote monitoring, communication and training, disease and outbreak tracking, and support for diagnosis and treatment decision [54-56]. Based on this reasoning, we can conclude that there may be higher probabilities for individuals able to use and interact with eHealth effectively to having adequate or high health literacy, contrary to our finding (principal finding 2).

#### Principal Finding 3: Lower General Health Numeracy Predicted Susceptibility to Breast Cancer Misinformation Among Chinese Female Patients, Especially for Those Aged 40 Years or Above Regardless of Education

Results of our study show that patients’ general health numeracy is an important predictor of the odds of their vulnerability to breast cancer misinformation. The role of general health numeracy in the health domain has been well documented in relevant studies. As proposed by Rothman et al [57], a great variety of health-related tasks, such as reading food labels, refilling prescriptions, measuring medications, interpreting blood sugars or other clinical data, and understanding health risks, rely on numeracy skills. Schwartz et al [58] found that the study informants’ numeracy was intimately associated with the accurate application of quantitative information about the benefit of mammography to their perceived risk of death. A similar study revealed that higher numeracy skills warrant more consistent interpretation of breast cancer risks [59]. The significance of numeracy was also ascertained in the findings of other studies that higher numeracy is correlated with an improved ability to interpret the benefits of treatments [57] and that patients’ inability to deal with basic probability and numerical concepts is related to poorer anticoagulation control [60]. In our study, limited general health numeracy skills predicted worse response outcomes, that is, limited breast cancer misinformation ability. This result confirms the results concerning the predicting role of general health numeracy skills reported in previous studies [57-60].

#### Principal Finding 4: Moderate Self-Assessment of General Disease Knowledge Significantly Predicted Female Patients’ Higher Abilities to Appraise Breast Cancer Misinformation

This finding of our study could be explained clinically. Two researchers of this study (ZX and ZD), who are medical professionals, reported that patients did not know how to select the correct health knowledge when being exposed to diversified sources of health knowledge, especially when the health knowledge was not evidence-based information but misconceptions or myths about diseases. Such unverified health knowledge could possibly contribute to poor health literacy, even though patients reported higher levels of self-assessed disease knowledge, as explained by these 2 researchers. They (ZX and ZD) also said that they frequently met with such cases in outpatient clinics or wards, where patients claiming to know about specific diseases quite well actually knew little, and much of their claimed knowledge turned out to be incorrect. This finding of our study aligns well with the finding by Lorini et al [61] that what people think they know does not always equal what they really know. Specifically, people are more likely to be overconfident (they think they know more than they really know) or underconfident (they think they know less than they really know) [61].

Our counterintuitive finding concerning the role of moderate self-assessment of general disease knowledge in predicting female patients’ higher abilities to appraise breast cancer misinformation is not in tune with the findings of some previous studies. For example, limited health literacy was found to be associated with less health knowledge [62]. Similarly, health literacy was discovered to be independently related to disease knowledge [63]. By the same token, patients with limited health literacy know less about their disease and treatment, therefore having fewer correct self-management skills, in comparison with literate patients [64,65]. These findings warrant future studies to verify and ascertain the role of moderate self-assessment of general disease knowledge attested in our study.

#### Principal Finding 5: More Positive Health Beliefs and Higher Levels of Self-Confidence Were Significant Predictors of Higher Abilities to Appraise Breast Cancer Misinformation Among Chinese Patients, Male and Female

Our most revealing finding was the role of MHLC Form A statement 6 (“I am in control of my health”) and statement 12 (“The main thing that affects my health is what I myself do”) in predicting participants’ abilities to appraise breast cancer misinformation. These predictors, more positive health beliefs and higher levels of self-confidence, have not been explored in the literature to the best of our knowledge and according to our retrieval of relevant studies. Therefore, we could not compare this finding with previous studies.

### Implications

This study can add to the body of evidence supporting the necessity of investigating the susceptibility to breast cancer misinformation. Important implications can be provided in terms of clinical practice, health education, medical research, and public health policy making. The 5 significant predictors of breast cancer misinformation susceptibility identified in the study could be used as important indicators for screening those susceptible to breast cancer misinformation in order to deliver targeted education and interventions. Knowledge and skills related to the 5 predictors should be integrated into public health education about breast cancer misinformation in order to improve the general public’s ability to appraise and rebut breast cancer myths. Medical researchers may gain insights into the topic of the susceptibility to breast cancer misinformation. As a result, they could verify the contributors to breast cancer misinformation susceptibility ascertained in this study and identify more contributing factors in future studies. Public health policy makers can consider the results and findings of this study when making public health policies in the future.

### Limitations

This study has some limitations. The first limitation is concerned with data collection. We recruited patients from only 1 hospital in Shandong Province, China, so the data collected may not represent the whole Chinese population. As a result, the results and principal findings of this study may not be completely generalized to the overall Chinese population. As Shandong Province is a densely populated province with relatively low socioeconomic development, the collected data may represent people in other provinces with similar population and socioeconomic characteristics. Second, we cannot sufficiently explain principal finding 5, because only a limited number of relevant studies could be retrieved from the existing literature. The third limitation is related to the predicting role of the self-assessment of general disease knowledge. This predictor played different roles in identifying susceptibility among participants with different educational attainments, as explained in principal finding 4. Further studies are needed to ascertain the causes underlying this “tricky” predictor.

### Conclusion

Drawing on logistic regression modeling, we studied the susceptibility to breast cancer misinformation among Chinese patients. We found that women’s limited ability to rebut breast cancer misinformation is mainly attributed to 5 factors: (1) lower communicative health literacy, (2) certainty about self-assessed eHealth literacy skills, (3) lower general health numeracy, (4) positive self-assessment of general disease knowledge, and (5) more negative health beliefs and lower levels of self-confidence. These predicting factors of the susceptibility to breast cancer misinformation can provide insightful implications for clinical practice, health education, medical research, and health policy making.

## Appendix

Multimedia Appendix 1 Myths about breast cancer.

Multimedia Appendix 2 Descriptive statistics of the study participants.

## Abbreviations

AAHLS: All Aspects of Health Literacy Scale
eHEALS: eHealth Literacy Scale
GHNT-6: General Health Numeracy Test
MHLC: Multidimensional Health Locus of Control (MHLC) Scales
OR: odds ratio
VIF: variance inflation factor

## Data Availability

Data are available upon reasonable request from the corresponding author.
