# Supplementary material for: Susceptibility to Breast Cancer Misinformation Among Chinese Patients: Cross-sectional Study
Source: JMIR Form Res. 2023 Apr 5;7:e42782. doi: 10.2196/42782 (PMC10131805; doi:10.2196/42782)
Supplement: Multimedia Appendix 1 [file formative_v7i1e42782_app1.docx]

**Appendix 1. Myths about breast cancer.**

| 1 | Finding a breast lump means you have breast cancer. |
| --- | --- |
| 2 | Breast cancer can always cause you to feel a lump. |
| 3 | Men do not get breast cancer; it only affects women. |
| 4 | If you have a family history of breast cancer, you are also likely to develop breast cancer. |
| 5 | If you maintain a healthy weight, exercise regularly, eat a healthy diet and limit your alcohol consumption, you don't have to worry about breast cancer. |
| 6 | Wearing a bra can lead to breast cancer. |
| 7 | An annual mammogram will ensure early detection of breast cancer. |
| 8 | Early-stage breast cancer rarely recurs. |
| 9 | Breast cancer only occurs in middle-aged and older women. |
| 10 | You will be free of breast cancer after your treatment is over. |

Sources of myths:

https://www.breastcancertrials.org.au/common-breast-cancer-myths-and-facts/

https://www.breastcancer.org/facts-statistics/myths-vs-facts

<https://www.nationalbreastcancer.org/breast-cancer-myths/>
