# Supplementary material for: Susceptibility to Breast Cancer Misinformation Among Chinese Patients: Cross-sectional Study
Source: JMIR Form Res. 2023 Apr 5;7:e42782. doi: 10.2196/42782 (PMC10131805; doi:10.2196/42782)
Supplement: Multimedia Appendix 2 [file formative_v7i1e42782_app2.docx]

**Appendix 2. Descriptive statistics of the study participants.**

| **Variables** | **Minimum** | **Maximum** | **Mean** | **Std. Deviation** |
| --- | --- | --- | --- | --- |
| Age | 17 | 66 | 38.29 | 11.52 |
| Gender (female %) | 77.85% | | | |
| Education | 1 | 6 | 3.68 | 1.46 |
| Self-Assessed Disease Knowledge | 1 | 4 | 2.50 | 0.92 |
| FHL1 | 1 | 3 | 2.12 | 0.72 |
| FHL2 | 1 | 4 | 1.96 | 0.91 |
| FHL3 | 1 | 3 | 2.15 | 0.73 |
| FHL_SUM | 3 | 10 | 6.22 | 1.34 |
| COHL1 | 1 | 3 | 1.60 | 0.73 |
| COHL2 | 1 | 3 | 1.82 | 0.73 |
| COHL3 | 1 | 3 | 1.79 | 0.74 |
| COHLSUM | 3 | 9 | 5.22 | 1.54 |
| CRHL1 | 1 | 3 | 1.95 | 0.75 |
| CRHL2 | 1 | 3 | 1.87 | 0.69 |
| CRHL3 | 1 | 3 | 1.87 | 0.72 |
| CRHL4 | 1 | 3 | 2.03 | 0.73 |
| CRHL5 | 1 | 3 | 1.96 | 0.72 |
| CRHL6 | 1 | 2 | 1.51 | 0.50 |
| CRHL_SUM | 6 | 17 | 11.19 | 1.99 |
| eHL1 | 1 | 5 | 2.92 | 1.11 |
| eHL2 | 1 | 5 | 3.02 | 1.12 |
| eHL3 | 1 | 5 | 3.06 | 1.12 |
| eHL4 | 1 | 5 | 3.09 | 1.09 |
| eHL5 | 1 | 5 | 3.06 | 1.18 |
| eHL6 | 1 | 5 | 3.00 | 1.16 |
| eHL7 | 1 | 5 | 3.10 | 1.11 |
| eHL8 | 1 | 5 | 2.97 | 1.14 |
| eHLSUM | 8 | 40 | 24.21 | 5.49 |
| GHNT1 | 1 | 2 | 1.57 | 0.49 |
| GHNT2 | 1 | 2 | 1.21 | 0.41 |
| GHNT3 | 1 | 2 | 1.24 | 0.43 |
| GHNT4 | 1 | 2 | 1.90 | 0.30 |
| GHNT5 | 1 | 2 | 1.82 | 0.39 |
| GHNT6 | 1 | 2 | 1.73 | 0.44 |
| GHNT_SUM | 6 | 12 | 9.47 | 1.27 |
| MHLC_A1 | 1 | 6 | 3.37 | 1.49 |
| MHLC_A6 | 1 | 6 | 3.87 | 1.66 |
| MHLC_A8 | 1 | 6 | 3.22 | 1.44 |
| MHLC_A12 | 1 | 6 | 3.63 | 1.51 |
| MHLC_A13 | 1 | 6 | 3.44 | 1.50 |
| MHLC_A17 | 1 | 6 | 3.66 | 1.55 |
| MHLC_SUM | 6 | 36 | 21.19 | 5.63 |
| Myth1 | 1 | 2 | 1.27 | 0.44 |
| Myth2 | 1 | 2 | 1.43 | 0.49 |
| Myth3 | 1 | 2 | 1.25 | 0.43 |
| Myth4 | 1 | 2 | 1.58 | 0.49 |
| Myth5 | 1 | 2 | 1.42 | 0.49 |
| Myth6 | 1 | 2 | 1.28 | 0.45 |
| Myth7 | 1 | 2 | 1.67 | 0.47 |
| Myth8 | 1 | 2 | 1.57 | 0.50 |
| Myth9 | 1 | 2 | 1.24 | 0.43 |
| Myth10 | 1 | 2 | 1.32 | 0.47 |
| Myth_ SUM | 10 | 20 | 14.03 | 1.78 |
| Valid N | 447 |  |  |  |
